# A framework for automated speech and language analysis in Spanish‐speaking Latinos

**DOI:** 10.1002/alz.086973

**Published:** 2025-01-03

**Authors:** Adolfo M. Garcia

**Affiliations:** ^1^ Cognitive Neuroscience Center, University of San Andrés, Victoria, Buenos Aires Argentina

## Abstract

**Background:**

Automated speech and language analysis (ASLA) represents a powerful innovation for detecting and monitoring persons with or at risk for dementia. Given its cost‐efficiency and automaticity, its impact can be vital for under‐resourced communities, such Spanish‐speaking Latinos. However, ASLA markers are understudied in this group and may differ from those established in widely studied populations (e.g., English speakers). Here I will describe a novel framework to boost ASLA research among Spanish‐speaking Latinos.

**Method:**

We have created hypothesis‐driven metrics to capture semantic and episodic memory alterations via (semi‐) spontaneous speech in Latinos. Our initial work includes measures of word selection patterns during verbal fluency, speech timing proxies of word retrieval effort, and algorithms quantifying egocentric/exocentric references in discourse. These metrics have been incorporated into the TELL app, our web‐based speech testing platform. Initial analyses have been performed on 60 Latinos with Alzheimer’s disease (AD), 65 with mild cognitive impairment (MCI), and 50 with behavioral variant frontotemporal dementia (bvFTD), including tests of cross‐linguistic generalizability with English speakers.

**Results:**

Compared with healthy controls, AD and MCI (but not bvFTD) patients exhibit atypical vocabulary patterns during verbal fluency tasks, favoring high frequency and conceptually unspecific words, separated by longer pauses. These alterations discriminate between persons with and without these disorders (AD: AUC = .89; MCI: AUC = 81), and they predict atrophy of and hypo‐connectivity among temporo‐parietal regions implicated in semantic memory. Cross‐linguistic generalizability between English and Spanish‐speaking AD patients was maximal (AUC = .79) when based on speech timing features (e.g., pause duration, articulation rate). Finally, bvFTD (but not AD) patients showed abnormally exocentric discourse, with increased reliance on third‐person references and reduced reliance on first‐person references when describing daily activities.

**Conclusion:**

Our initial findings attest to the usefulness of hypothesis‐driven ASLA features to identify persons with AD, MCI, and bvFTD among the Latino population. The framework will now be leveraged in a large multicentric effort across the ReDLat consortium to establish the most robust markers in large, heterogeneous samples. This work will help us streamline ASLA research as an avenue for more equitable clinical testing of dementia in Latin America and beyond.

